# Lysozyme-like Protein Produced by *Bifidobacterium longum* Regulates Human Gut Microbiota Using In Vitro Models

**DOI:** 10.3390/molecules26216480

**Published:** 2021-10-27

**Authors:** Mingzhu Du, Xinqiang Xie, Shuanghong Yang, Ying Li, Tong Jiang, Juan Yang, Longyan Li, Yunxiao Huang, Qingping Wu, Wei Chen, Jumei Zhang

**Affiliations:** 1 State Key Laboratory of Food Science and Technology, Jiangnan University, Wuxi 214122, China; 6190112018@stu.jiangnan.edu.cn (M.D.); 6180112106@stu.jiangnan.edu.cn (S.Y.); chenwei66@jiangnan.edu.cn (W.C.); 2 School of Food Science and Technology, Jiangnan University, Wuxi 214122, China; 3 Guangdong Provincial Key Laboratory of Microbial Safety and Health, State Key Laboratory of Applied Microbiology Southern China, Institute of Microbiology, Guangdong Academy of Sciences, Guangzhou 510070, China; liying@gdim.cn (Y.L.); jt0925@stu.scau.edu.cn (T.J.); yj18185238563@163.com (J.Y.); 18868006204@126.cn (L.L.); wuqp203@163.com (Q.W.); 4 College of Life Sciences, South China Agricultural University, Guangzhou 510642, China; hyxiao2000@icloud.com

**Keywords:** *Bifidobacterium longum*, lysozyme-like protein, bioinformatics, bioactivity, microbiome, balance

## Abstract

The extracellular secreted protein of *Bifidobacterium longum* (*B. longum*) plays an important role in maintaining the homeostasis of the human intestinal microenvironment. However, the mechanism(s) of interaction remain unclear. Lysozyme is a kind of antibacterial peptide. In this study, the amino acid sequence of a lysozyme-like protein of *B. longum* based on whole-genome data of an isolate from human gut feces was found. We further predicted functional domains from the amino acid sequence, purified the protein, and verified its bioactivity. The growth of some bacteria were significantly delayed by the 020402_LYZ M1 protein. In addition, the gut microbiota was analyzed via high-throughput sequencing of 16S rRNA genes and an in vitro fermentation model, and the fluctuations in the gut microbiota under the treatment of 020402_LYZ M1 protein were characterized. The 020402_LYZ M1 protein affected the composition of human gut microbiota significantly, implying that the protein is able to communicate with intestinal microbes as a regulatory factor.

## 1. Introduction

Each area of the human gut represents a habitat with unique structure, chemical [1], and biological components [2], which is similar to the river system to some extent [3]. There are trillions of microorganisms [4] and hundreds of distinct bacterial species in the gut—some pathogenic and some beneficial [5]. Millions of years of co-evolution between host and its intestinal microbial ecosystem has formed a symbiotic relationship [6]. Disruptions in gut homeostasis are associated with massive disorders including those affecting circadian rhythmicity, nutritional responses, intestinal diseases, and some metabolic disorders [7], which suggests that the balance of intestinal microbial ecology is crucial for maintaining a healthy immune system [8,9,10]. *B. longum* were purported to exert various promoting effects in intestinal dynamic balance and host health [11,12,13], but the specific mechanism is still obscure. These studies uncovered various molecules that act as important mediators for the establishment of a *Bifidobacteria*—host communication, such as pili [14], extracellular polysaccharides [15], serpins, TagA [16], BopA [17], etc.

Microbial lysozyme is a kind of hydrolase, also known as muramidase or *N*-acetylmuramide glycanohydrolase. The efficiency of lysozyme in inhabiting gram-positive bacteria depends on its capacity to hydrolyze the β-(1,4)-glycosidic bond between *N*-acetylmuramic acid and *N*-acetylglucosamine [18]. The breakage of the peptidoglycan skeleton structure results in cell wall damage, and then the bacteria will be ruptured due to the imbalance of osmotic pressure, which leads to the lysis and death of the bacterial cells. The earliest research about the production of lysozyme by microorganisms can be traced back to Nikole’s report on the lysis factor of *Bacillus subtilis* (*B. subtilis*) [19,20]. Subsequently, Fleming discovered a special enzyme in 1922, which came from human saliva and tears [21], and named "lysozyme" because of its lytic ability to dissolve bacterial cell walls. Recently, some research has linked lysozyme with immunity and disease processes [22,23]. Yu showed that Paneth cell lysozyme regulates the relative abundance of mucolytic commensal bacteria and thereby the intestinal inflammatory response [24]. Zhou confirmed that *B. longum* enhanced mucosal repair, promoted the production of lysozyme, reduced the relative abundance of various pathogens, such as *Bacteroidales*, *Alloprevotella* and *Bacteroides,* and further ameliorated the dysbiosis of the microbiota in WAS rats [25]. These results all reflected the fact that *Bifidobacterium* maintained the stability of host health and intestinal homeostasis by promoting the secretion of lysozyme came from host cells. Simultaneously, Wu found that lysozyme affected the microbial composition and quality of grass carp flakes stored at 4 °C [26], which was consistent with the previous research. In order to prevent the premature release of lysozyme in the stomach, Zhang designed a system targeting delivery of lysozyme to the intestine directly [27], which made sure that lysozyme could work normally. It is noteworthy that this study discovered a gene encoding a lysozyme-like protein in the whole-genome data of *B. longum*.

In this study, we aimed to explore the role of lysozyme-like protein and its impact on the gut microbiome. Bioinformatic methods were used to analyze the sequence of lysozyme-like protein, followed by expression and purification of that protein subsequently. Functions of the protein were probed by high-throughput sequencing of 16S rRNA and an in vitro model. The results of this study emphasized the lysozyme-like protein derived from *B. longum* and its bioactivity. Besides, the protein modulated the composition of the human gut microbiome, which better understood the specific mechanisms that *B. longum* involved in gut microbiota modulation.

## 2. Results and Discussion

### 2.1. Bioinformatic Analysis of Lysozyme-Like Protein

**Figure 1:**
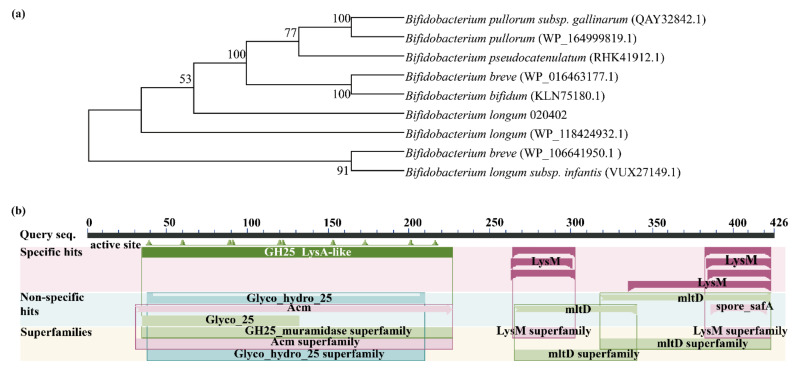
Homology and functional prediction of lysozyme-like protein. (**a**) Phylogenetic tree of lysozyme-like protein sequence. (**b**) Conserved domains of lysozyme-like protein.

The sequence encoding a lysozyme-like protein numbered 020402_00271 was found by analyzing the signal peptide and transmembrane domain of the whole-genome data of *B. longum* 020402. Molecular phylogenetic tree showed that lysozyme-like protein had high sequence homology in different strains, and their evolutionary relationship is relatively close. Besides, 020402_00271 and WP118424932.1 came from the same species (Figure 1a) where the amino acid sequence similarity was 100% (Figure S1). Based on the Conserved Domain database, the prediction showed that the 020402_00271 protein belongs to the GH25_muramidase superfamily, GH25 superfamily, Acm superfamily, LysM superfamily, and mltD superfamily (Figure 1b). The N-terminal glycosyl hydrolase family 25 (GH25) domain of LysA has sequence similarity with other murein hydrolase catalytic domains, which degrades bacterial cell walls by catalyzing the hydrolysis of 1,4-beta-linkages between *N*-acetylmuramic acid and *N*-acetyl-d-glucosamine residues [28,29]. In addition, Acm participated in cell wall/membrane/envelope biogenesis [30], LysM is a widely distributed protein motif for binding to (peptido) glycans [31], and MltD plays a role in muropeptides recycling during cell elongation and/or cell division [32].

### 2.2. Structure and Purification of 020402_LYZ M1 Protein

**Figure 2:**
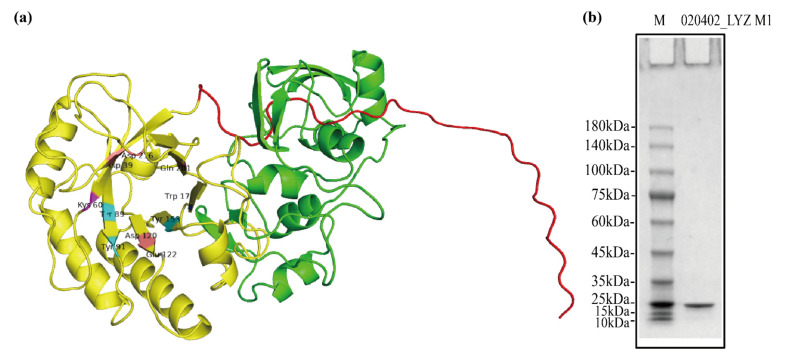
Tertiary structure prediction and gel map. (**a**) Homology analysis and model prediction of the 020402_00271 protein. (**b**) The SDS-PAGE analysis of 020402_LYZ M1 protein, M: marker.

The signal peptide (1–33 amino acids) of 020402_00271 protein was predicted by using SignalP 5.0 and TMHMM 2.0. Besides, the prediction of tertiary structure indicated that the 020402_00271 protein corresponded to 1,4-beta-*N*-Acetylmuramidase M1 (Figure 2a and Figure S2). The value of GMQE was 0.32 and QMEAN was 0.62 ± 0.06 in this model, which proved the result was reasonable. By comparing sequences between the 020402_00271 protein and template protein (1jfx.1.A), the length of former sequence was found longer than that of 1jfx.1.A (Figure S3). The extra amino acids of the 020402_00271 protein were simultaneously highlighted in green (Figure 2a and Figure S4). Two pairs of primers were used to amplify the gene encoding a protein without signal peptides (34–426 amino acids) and encoding GH25 LysA-like domain (34–234 amino acids), respectively, followed by vector construction, protein expression, and purification subsequently. Finally, only one protein (34–234 amino acids) was purified successfully and named 020402_LYZ M1. Besides, SDS-PAGE showed a clear and single band (Figure 2b), which meant the 020402_LYZ M1 protein was pure.

The molecular mass of the 020402_LYZ M1 protein coded for 234 amino acid residues was estimated at 25.84 kDa, which corresponded to the result of SDS-PAGE. According to the analysis of Phyre2 online software, the 020402_LYZ M1 protein had three binding sites, and its residues were D39, W43, D120, E122, Y153, A175, Y177, Q201, S204, T205, N214, D216, E122, S123, Q155, A156, A175, Q176, Y177, W149, K169, E197, Q226, A229, and Y230. In addition, the active sites of this protein were D39, K60, Y89, Y91, D120, E122, Y153, W173, Q201, and D216 (Figure 2a). Both binding sites and active sites were conservative highly in the 020402_LYZ M1 protein (Figure S5), which meant the 020402_LYZ M1 protein maintained the functions of these conserved domains.

### 2.3. Bioactivity of 020402_LYZ M1 Protein

**Figure 3:**
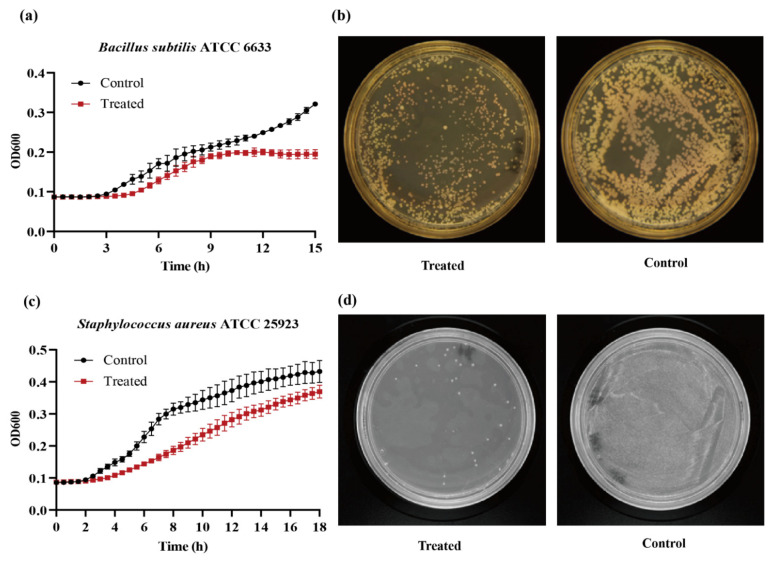
Bioactivity of the 020402_LYZ M1 protein. (**a**) The growth curves of *B. subtilis* ATCC 6633. (**b**) The dilution coating plate experiment of *B. subtilis* ATCC 6633. (**c**) The growth curves of *S. aureus* ATCC 25923. (**d**) The dilution coating plate experiment of *S. aureus* ATCC 25923.

The inhibition of bacterial growth by B17_2 was explored using two gram-positive strains, *B. subtilis* ATCC 6633 and *S. aureus* ATCC 25923. The time-inhibition curve could provide information about the kinetics of bacterial growth [33]. As for *B. subtilis* ATCC 6633, the curve of the control group showed a significant increase after 3 h. However, the experiment group treated with the 020402_LYZ M1 protein went up from 4 h to 9 h, reaching a plateau period after 9 h (Figure 3a), which indicated that 020402_LYZ M1 protein delayed the logarithmic growth of *B. subtilis* ATCC 6633 for one hour. The bacterial suspension of *B. subtilis* ATCC 6633 cultured for 9 h were cultured onto MRS agar plates. Compared with the control group, the results of group treated with 020402_LYZ M1 showed the same tendency, with fewer viable bacteria (Figure 3b). Similarly, 020402_LYZ M1 also affected the growth of *S. aureus* ATCC 25923. Although the two groups entered the logarithmic growth phase after 2 h of fermentation, the growth curve of the control group rose more significantly than that of treated with 020402_LYZ M1 (Figure 3c). Dilution coating plate experiments were also carried out with *S. aureus* ATCC 25923 cultivated for 9 h. The trend was the same as the time-inhibition curve, where the number of viable bacteria reduced sharply with 020402_LYZ M1 treatment (Figure 3d). These results all illustrated that 020402_LYZ M1 had antibacterial bioactivity; that is, it only delayed bacterial growth, which is consistent with the discovery of Derde [34].

### 2.4. 020402_LYZ M1 Protein Regulates Gut Microbiota

**Figure 4:**
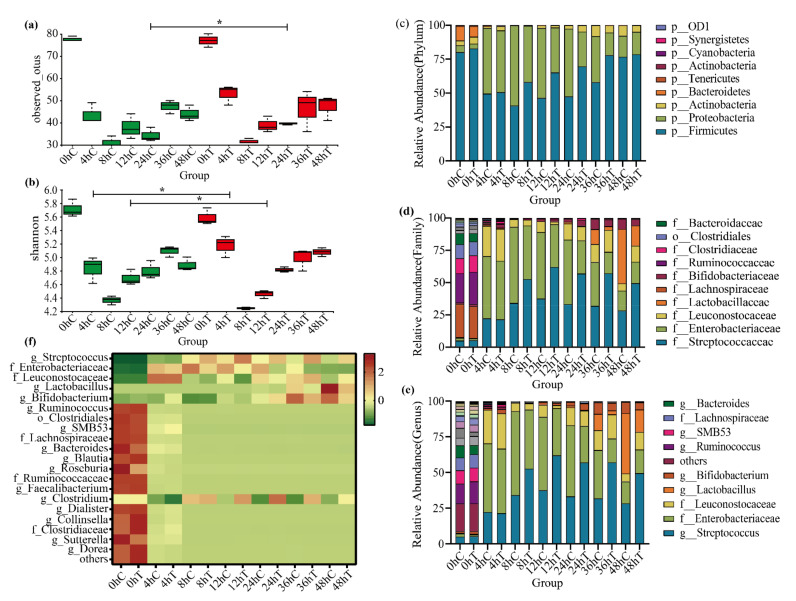
The 020402_LYZ M1 protein modulates the composition of gut microbiota. The numbers of replicated samples in this figure are as follows: 0 h (*n* = 3), 4 h (*n* = 3), 8 h (*n* = 3), 12 h (*n* = 3), 24 h (*n* = 3), 36 h (*n* = 3), 48 h (*n* = 3); C: control group; T: treatment group. (**a**) Observed Otus. (**b**) Shannon index. (**c**) Top eight of the relative abundances of gut microbiota at Phylum level. (**d**) Top ten at Family level. (**e**) Top ten at Genus level. (**f**) Heatmap at Genus level. * *p* < 0.05, *T*-test.

In order to verify the ablity of the 020402_LYZ M1 protein to regulate the intestinal microbiome, seven time points were selected to track the change of composition of the human intestinal microbiome. Observed OTUs are equal to the number of species in each sample: the more OTUs, the greater the number of species [35]. The Shannon index was used to estimate the microbial diversity in a sample: the larger the Shannon value, the higher the community diversity [36]. Compared with 0 h fermentation time, the observed OTUs and Shannon index were reduced at each time point after using TPY medium, which indicated that the single medium (TPY broth) cultivated all bacteria unsuccessfully and caused the loss of specific bacteria. Similar to the study by Namai, the use of a single medium could lead to the disappearance of a large number of gut microbes [37], which is a limitation that should be overcomed in future studies. When it comes to α-diversity, the species diversity of feces notably decreased after treatment with the 020402_LYZ M1 protein for 24 h. However, there was no significant difference between the treatment group and the control group after 48 h, showing that the 020402_LYZ M1 protein could not kill microorganisms and only delayed the growth of certain bacteria for a period of time (Figure 4a). It is noteworthy that the community diversity of gut microbes flustered throughout the whole fermentation process. In addition, the Shannon index of the experimental group treated with the 020402_LYZ M1 protein for 48 h increased, while the species diversity of the control group decreased after 48 h of fermentation, which indicated that the 020402_LYZ M1 protein had the ability to maintain species diversity (Figure 4b).

The results of high-throughput sequencing of 16S rRNA genes showed that the microbial communities of all samples covered 9 phyla, 14 classes, 25 orders, 55 families, and 80 genera. The gut microbiota compositions at the phylum, family, and genus levels were characterized as follows: at phylum level, the gut microbiotas fluctuated obviously from 8 h to 36 h. Compared with the control group, the relative abundance of *Firmicutes* increased in the group treated with 020402_LYZ M1 protein during the whole period, expect for 0 h and 48 h, while the relative abundance of *Proteobacteria* decreased. In contrast, the gap in the relative abundance of *Proteobacteria* was not significant between the treatment group and the control group by the end of the period, which indicated that the 020402_LYZ M1 protein could inhibit the colonization and expansion of the *Proteobacteria* within a certain period (Figure 4c).

When it comes to family level, the top ten of the gut microbial relative abundances can be listed as follows: *Streptococcaceae*, *Enterobacteriaceae*, *Leuconostocaceae*, *Lactobacillaceae*, *Lachnospiraceae*, *Bifidobacteriaceae*, *Ruminococcaceae*, *Clostridiaceae*, *Clostridiales*, and *Bacteroidaceae*. The relative abundance of Streptococcaceae increased gradually, while the relative abundance of *Enterobacteriaceae* decreased after treatment with the 020402_LYZ M1 protein, which was consistent with the tendency of phylum-level. Besides, the relative abundance of *Lactobacillaceae* reached the peak in 36 h, showing that Lactobacillaceae had entered the logarithmic growth phase after 36 h, which had a guiding significance of isolation and cultivation of *Lactobacillaceae* in the future. However, the relative abundance of *Lactobacillaceae* decreased after treatment with the 020402_LYZ M1 protein (Figure 4d).

At the genus level, the relative abundance of *Streptococcus* ranked first, expect for 0 h (Figure 4e). Compared with the relative abundances between treatment group and the control group at this level, the relative abundances of *Streptococcus*, *Leuconostocaceae*, and *Clostridiaceae* in the former group increased, while the relative abundances of *Lactobacillus* and *Bifidobacterium* decreased. Besides, the same trends were showed in a cluster heat map (Figure 4f). It is common that *Bifidobacterium* and *Lactobacillus* are beneficial to human health, however, some studies showed that Bifidobacterium and Lactobacillus were risk factors in the fecal microbiota transplantation (FMT) and lactose intolerance (LI) [38,39,40,41]. Treatment of LI can include a low-lactose diet [42], lactase supplementation [43], probiotics [44], and colonic adaptation by prebiotics [45], such as galacto-oligosaccharides (GOS) [46], in which lactase is used to hydrolyse lactose into galactose and glucose to alleviate lactose intolerance symptoms. It is interesting that the kinds of protein added to dairy products are widely available and considered safe. However, residual side proteolytic activity of lactase can degenerate casein and impair taste, especially after long storage [47]. As for the impact of the gut microbiome on the occurrence of gut-related LI symptoms, Gois provided evidence that specific gut symptoms, experienced by lactose intolerance (LI) patients, might be the result of *Bifidobacterium* abundance in the gut, rather than a direct effect of lactose intake [41], which supports initial reports where metabolic products of lactose-fermenting bacteria may be related to LI symptom occurrence [48,49,50]. Notably, the abundance of *Bifidobacterium* decreased by using the 020402 LYZ_M1 protein in this study, which inspired us to investigate whether the ability of the 020402 LYZ_M1 protein affects the *Bifidobacterium* abundance in LI individuals. These results reflected that the 020402 LYZ_M1 protein was involved in gut microbiota modulation in this study. Simultaneously, a healthy gut microbiome relies on high richness and biodiversity [51]. If the dominant positions of the bacteria reported as probiotics are emphasized blindly, the balance of intestinal microecology will be broken.

### 2.5. 020402_LYZ M1 Protein Restrain the Growth of Lactobacillus

**Figure 5:**
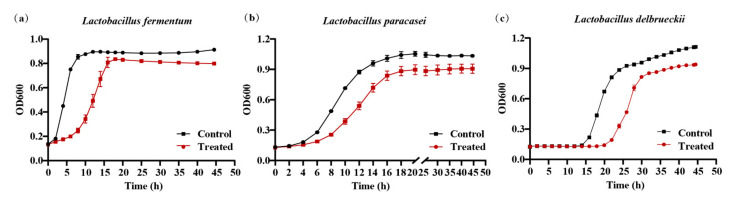
020402_LYZ M1 affects the growth of *Lactobacillus*. (**a**) Growth curve of *Lactobacillus fermentum*. (**b**) Growth curve of *Lactobacillus paracasei*. (**c**) Growth curve of *Lactobacillus delbrueckii*.

The flora maintains the steady state of the microenvironment with their proportions in the intestine respectively. In order to verify the ability of the 020402_LYZ M1 protein to inhibit the overgrowth of *Lactobacillus*, *Lactobacillus fermentum, Lactobacillus paracasei,* and *Lactobacillus delbrueckii*, three strains were isolated from the samples from the in vitro fermentation experiment and treated with the 020402_LYZ M1 protein. Compared with the control group, the 020402_LYZ M1 protein reduced the bacterial mass and delayed the time to enter the logarithmic phase of *L. fermentum, L. paracasei*, and *L. delbrueckii* for 4 h, 6 h, and 5 h, respectively (Figure 5a–c). These conclusions corresponded to the previous experiment, where the 020402_LYZ M1 protein reduced the relative abundance of *Lactobacillus* and inhibited the excessive growth of *Lactobacillus* (Figure 4f).

## 3. Materials and Methods

### 3.1. Bacterial Isolates and Sequencing

*B. longum* 020402 was isolated from human fecal samples, coming from Jiaoling, Guangdong, China. Bacterial DNA was extracted by HiPure Microbial DNA Kit (Magen Biotech, Guangzhou, China) according to the manufacturer’s instructions. Its whole-genome data were obtained by using the Illumina NextSeq instrument owned by the Institute of Microbiology, Guangdong Academy of Sciences.

### 3.2. Analysis of Sequence Homology

In the National Center for Biotechnology Information (NCBI), https://www.ncbi.nlm.nih.gov/(accessed on 8 August 2021), Basic Local Alignment Search Tool (BLASTp) was used to search for homologous sequences of protein in different species and MEGAX software (Mega Limited, Auckland, New Zealand)was used to construct a Neighbor-Joining evolutionary tree with 1000 bootstraps, and then the following tools were used for analysis: CLUSTALW https://www.genome.jp/tools-bin/clustalw (accessed on 8 August 2021)for sequence alignment and ESPript https://espript.ibcp.fr/ESPript/cgi-bin/ESPript.cgi (accessed on 8 August 2021) for presentation of the results.

### 3.3. Analysis of Structure and Function

The following tools were used to predict each element: SignallP 5.0 http://www.cbs.dtu.dk/services/SignalP (accessed on 8 August 2021) for the N-terminal signal peptide prediction, TMHMM 2.0 http://www.cbs.dtu.dk/services/TMHMM (accessed on 8 August 2021) for transmembrane region prediction, and ProtParam https://web.expasy.org/protparam (accessed on 8 August 2021) for physical and chemical properties prediction. Besides, Conservative domain database Home—Conserved Domains—NCBI (nih.gov) (accessed on 15 August 2018) was used to predict functional domains. The three-dimensional structures and the binding sites of the protein were predicted by using SWISS-MODEL https://swissmodel.expasy.org (accessed on 10 August 2021) and phyre2 http://www.sbg.bio.ic.ac.uk/phyre2 (accessed on 11 August 2021) accessed on respectively. PyMOL version 2.5.0 was used to analyze the structural modeling.

### 3.4. Gene Cloning of Target Protein

The whole-genome DNA extracted from *B. longum* 020402 was used as a template, and two pairs of primers were designed to clone the target gene. The gene encoding a protein without signal peptide (34–426 amino acids) was cloned by using primer pair 1 (F: 5′-AAAAAACATATGGTGGCGGATATGCAGGGCATTG-3′; R: 5′-GTGGTGCTCGAGTCGGTAGTGCAGCACTTCGCCGGG-3′). The gene encoding GH25 LysA-like domain (34–234 amino acids) was amplified by using primer pair 2 (F: 5′-AATTATCCATGGCAGTGGCGGATATGCAGGGCATTG-3′; R: 5′-GTGGTGCTCGAGGTCGCCCTGCGCGTACGCGTC-3′). The resulting gene fragment and plasmid pET28a (+) were digested with restriction endonucleases (NEB, Ipswich, MA, USA), and then the target fragment was recovered by using purification kit (Omega bio-tek, Norcross, GA, USA) and gel recovery kit (Omega bio-tek, Norcross, GA, USA). After that, the target fragment and the plasmid were ligated by using T4 ligase (NEB, Ipswich, MA, USA) and subsequently transformed into *Escherichia coli* (*E. coli*) DH5α (Sangon Biotech, Shanghai, China). The positive colons were screened by amplification with primer pair (T7: 5′-TAATACGACTCACTATAGGG-3′; T7-Term: 5′-GCTAGTTATTGCTCAGCGG-3′) and Sanger sequencing. If the target gene had no base mutations, the vector of pET-28a (+)/target gene was constructed successfully.

### 3.5. Heterologous Expression and Purification of Target Protein

Next, the target protein overexpression was induced in *E. coli* BL21 (DE3) (Sangon Biotech, Shanghai, China). Afterwards, the cells were collected by centrifugation (10,000× *g*, 20 min, 4 °C), dissolved in 35 mL of lysis buffer (1M NaCI, 50 mM phosphate buffer saline, 70 mM Imidazole, 1 mg/mL lysozyme, pH = 7.6), and stored at −80 °C until use.

According to Xie’s methods [52], we took out the bacterial cells stored at −80 °C, followed by ultrasonic fragmentation at 0 °C. Then, the supernatant was harvested by centrifugation (15,000× *g*, 30 min, 4 °C), filtrated by 0.8 μm filters, and stored at 4 °C until use. The filtrated supernatant was loaded on a 5 mL His Trap nickel column (HisTrap TM FF, GE Healthcare, Chicago, IL, USA) pre-equilibrated with the lysis buffer (1M NaCI, 50 mM phosphate buffer saline, 70 mM Imidazole, pH = 7.6). After that, the target protein with His-tag were collected by washing buffer (150 mM NaCI, 50 mM phosphate buffer saline, 200 mM Imidazole, pH = 7.2). In order to concentrate the target protein and remove salt ions from that, the collected protein was transferred to a MWCO 10 KDa ultrafiltration tube (Millipore, Burlington, MA, USA), mixed with exchange buffer (100 mM NaCI, 50 mM phosphate buffer saline, 10 % glycerin, pH = 7.2), and centrifuged (4000 rpm, 40 min, 4 °C), which was repeated twice. Finally, the target protein was mixed with 20% glycerol, divided into 1.5 mL tubes, and stored at −80 °C until use. Lysis buffer, ddH_2_O, EDTA solution, ddH_2_O, nickel sulfate, and ddH_2_O were subsequently used to clean the nickel column, which was finally stored at 4 °C. Sodium dodecyl sulfate–polyacrylamide gel electrophoresis (SDS-PAGE) was used to verify the purity of the target protein.

### 3.6. Evaluation of Bioactivity

The bacterial cells of *Bacillus subtilis* ATCC 6633, *Staphylococcus aureus* ATCC 25923, *Lactobacillus fermentum*, *Lactobacillus paracasei*, and *Lactobacillus delbrueckii* were collected, when reaching to the logarithmic phase. Then, 10^6^ CFU/mL bacterial suspensions were prepared. After that, 2.5% 020402_LYZ M1 protein or a mixture of exchange buffer (100 mM NaCI, 50 mM phosphate buffer saline, 10% glycerin, pH = 7.2) with 20% glycerol were administered to the treatment groups and the counterparts respectively, and all groups were incubated at 37 °C for 48 h. The OD_600_ reading was measured by using a microplate reader, and three parallel entries of the kinetic growth information were recorded every 30 min. Time-inhibition curves were derived to identify the time point at which inhibition was observed after 020402_LYZ M1 protein treatment, and dilution coating plate experiment was used to observe the number of viable bacteria. In addition, the following media were used for bacterial culturing: MRS medium (Huankai, Guangzhou, China) for *Bacillus subtilis* ATCC 6633, LB medium (Huankai, Guangzhou, China) for *Staphylococcus aureus* ATCC 25923, TPY medium (Hopebio, Qingdao, China) for *Lactobacillus fermentum*, *Lactobacillus paracasei*, and *Lactobacillus delbrueckii.*

### 3.7. In Vitro Fermentation Experiment and Illumina MiSeq Sequencing

A stool sample was collected from a healthy donor via fecal microbiota transplantation (FMT), which was approved by the ethics committee of the First Affiliated Hospital of Guangdong Pharmaceutical University (reference 2017-98) [53]. About 0.5 g of feces were weighed and placed on ice, then resuspended with 50 mL 0.9% stroke-physiological saline solution (SPSS). 1% of the mixture was inoculated into TPY broth after mixing well and the culture medium containing feces was divided into two parts, the treatment group (T) and the control group (C), with three parallel entries for each group. The treatment group was administered 2.5% of 020402_LYZ M1 protein, and the control group was treated with 2.5% of a mixture of exchange buffer (100 mM NaCI, 50 mM phosphate buffer saline, 10% glycerin, pH = 7.2) with 20% glycerol. Samples were collected at different ferment times (0 h, 4 h, 8 h, 12 h, 24 h, 36 h, and 48 h), centrifuged to remove the supernatant, and divided into two shares evenly. One share of the sample was mixed with 30% glycerol and stored at −80 °C until use, and the other sample were stored in dry ice and sent to a commercial laboratory (Genewiz, Inc., Suzhou, China) for high-throughput sequencing of 16S rRNA genes. The DNA templates were extracted from each sample, the V4-V5 region of the 16S rRNA genes were amplified, and the PCR products were sequenced by using MiSeq (Illumina, Inc., San Diego, CA, USA). The original binary base-calling data obtained by using sequencing were converted into pass filtering (PF) or raw sequence data by Illumina bcl2fastq software (Illumina, San Diego, CA, USA). Barcodes were removed from raw data by using Perl script and low-quality data were removed by using Trimmomatic version 0.39 [54].

### 3.8. Isolation of Lactobacillus

Based on in vitro ferment experiment described in Section 3.7, TPY medium was used for separation and cultivation of *Lactobacillus* from the samples stored at −80 °C. Firstly, the samples were diluted with 0.9% SPSS to obtain serial bacterial suspension (10-fold) from 10^−1^ to 10^−6^ dilution, 100 μL of that were cultured onto TPY agar plates until dry, and cultured with 3 parallels in anaerobic condition at 37 °C for 48 h. According to the different colonial morphologies, single colonies were selected randomly and streaked on TPY agar plates twice. After that, single colonies were inoculated into TPY broth until obvious turbidity was observed, collected, and identified by Sanger sequencing.

### 3.9. Statistical Analysis

*T*-test was used to evaluate the difference among all groups and the *p* values < 0.05 were considered significant. All data are presented in mean ± S.D. The graphic illustrations were generated with the GraphPad Prism 9 (GraphPad Software, San Diego, CA, USA).

## 4. Conclusions

This study characterized the amino acid sequence of a lysozyme-like protein of *B. longum* based on whole-genome data of an isolate from human gut feces. The sequence showed high homology with other of that and its GH25 LysA-like domain is highly conservative. Then, we expressed and purified the protein by using a gene encoding lysozyme-like protein partly, which was the bioactive protein with delaying the growth of some bacteria and modulating the composition of human gut microbiome in vitro. In summary, these results revealed the bioactivity of lysozyme-like protein and demonstrated changes in diversity of the human gut microbiome by using that from *B. longum*, which better understood the specific mechanisms that *B. longum* involved in gut microbiota modulation. Besides, it also provided a potential option for treatment of lactose intolerance. Future direction will include the exploration of targeted delivery of lysozyme-like protein and the proper resolution of lactose intolerance.

## Data Availability

Data will be available on the reasonable request from the corresponding author.

## Supplementary Materials

The following are available online, Figure S1: Multiple sequence alignment of lysozyme-like protein from nine *Bifidobacterium* strains, Figure S2: Prediction of tertiary structure of 020402_00271 protein by SWISS-MODEL, Figure S3: Sequence alignment between 020402_00271 protein and 1jfx.1.A, Figure S4: Amino acids sequence of 020402_00271 protein, Figure S5: Analysis of conserved sites of 020402_LYZ M1.
