# Supplementary material for: Lysozyme-like Protein Produced by Bifidobacterium longum Regulates Human Gut Microbiota Using In Vitro Models
Source: Molecules. 2021 Oct 27;26(21):6480. doi: 10.3390/molecules26216480 (PMC8587964; doi:10.3390/molecules26216480)
Supplement: Supplementary file 1 [file molecules-26-06480-s001.zip › molecules-1416405-supplementary.pdf]

**Figure S1.** Multiple sequence alignment of lysozyme-like protein from nine *Bifidobacterium* strains.

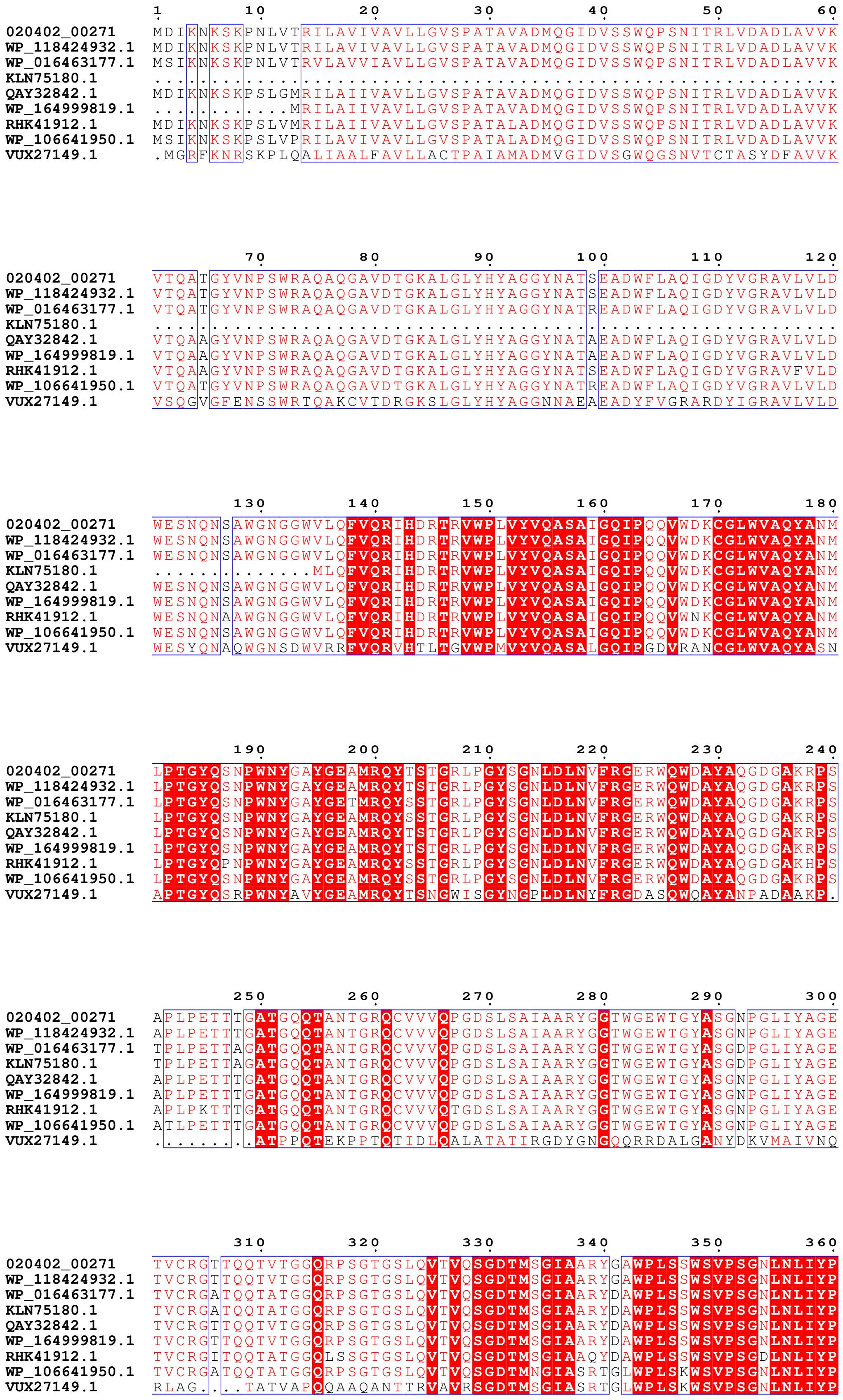

```
                          370       380       390       400       410       420
                            .         .         .         .         .         .
020402_00271      GQVVTYNGGGSVATGSNAPPATRTVTVRSGDTLSGIAARLGIGYTQLTGYRSGNPNVIYP
WP_118424932.1    GQVVTYNGGGSVATGSNAPPATRTVTVRSGDTLSGIAARLGIGYTQLTGYRSGNPNVIYP
WP_016463177.1    GQVVTYAGSYQYQSAG.....GRAYTVRSGDALSGIAARLGISWTQLTGYRSGNPSLIYP
KLN75180.1        GQVVTYAGSYQYQSAG.....GRAYTVRSGDTLSGIAARLGISWTQLTGYRSGNPSLIYP
QAY32842.1        GQVVTYTGAYQYQSAG.....GRAYIVRMGDTLSGIAQRLGVSMSQITGYRSGNPSLIYP
WP_164999819.1    GQVVTYTGAYQYQSAG.....GRAYIVRMGDTLSGIAQRLGVSMSQITGYRSGNPSLIYP
RHK41912.1        GQVVTYAGSYQYQSAG.....GRVYTVRSGDTLSGIAQRLGVSMSQITGYRSGNPSLIYP
WP_106641950.1    GQVVTYNGGGSVATGSNAPPATRTVTVRSGDTLSGIAARLGIGYTQLTGYRSGNPNVIYP
VUX27149.1        GQVVTYNGGGSVATGSN.APPSRTVTVRASASATRSSPAIAAATPT..............

020402_00271      GEVLHY
WP_118424932.1    GEVLHY
WP_016463177.1    GEVLYY
KLN75180.1        GEVLYY
QAY32842.1        GEVLRY
WP_164999819.1    GEVLRY
RHK41912.1        GEVLYY
WP_106641950.1    GEVLHY
VUX27149.1        ......
```

**Figure S2.** Prediction of tertiary structure of 020402_00271 protein by SWISS-MODEL.

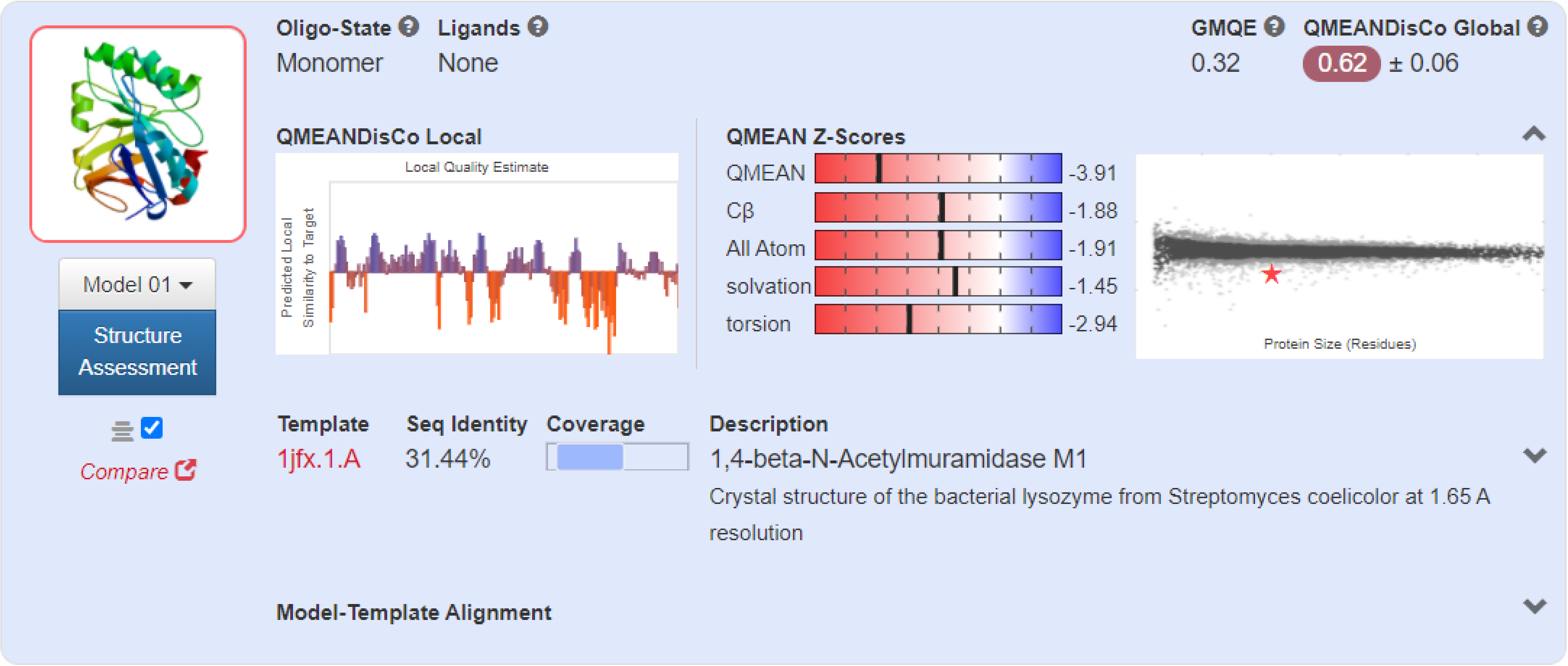

**Figure S3.** Sequence alignment between 020402_00271 protein and 1jfx.1.A.

```
               1        10        20        30        40        50
020402_00271   MDIKNKSKPNLVTRILAVIVAVLLGVSPATAVADMQGIDVSSWQPSNITRLV...DADLA
1jfx.1.A       ................................SGVQGIDVSHWQGSINWSSVKSAGMSFA

                60        70        80        90       100       110
020402_00271   VVKVTQATGYVNPSWRAQAQGAVDTGKALGLYHYAGGY..NATSEADWFLAQIGDY...V
1jfx.1.A       YIKATEGTNYKDDRFSANYTNAYNAGIIRGAYHFARPNASSGTAQADYFASNGGGWSRDN

                    120       130       140       150       160
020402_00271   GRAVLVLDWESNQN.......SAWGNGGWVLQFVQRIHDRTRVWPLVYVQASAIGQIPQ.
1jfx.1.A       RTLPGVLDIEHNPSGAMCYGLSTTQMRTWINDFHARYKARTTRDVVIYTTASWWNTCTGS

                    170       180       190       200       210       220
020402_00271   ..QVWDKCGLWVAQYANMLPTGYQSNPWNYGAYGEAMRQYTSTGRLPGYSGNLDLNVFRG
1jfx.1.A       WNGMAAKSPFWVAHWGVSAPT..VPSGFP....TWTFWQYSATGRVGGVSGDVDRNKFNG

                    230       240       250       260       270       280
020402_00271   ERWQWDAYAQGDGAKRPSAPLPETTTGATGQQTANTGRQCVVVQPGDSLSAIAARYGGTW
1jfx.1.A       SAARLLALAN..................................................

                    290       300       310       320       330       340
020402_00271   GEWTGYASGNPGLIYAGETVCRGTTQQTVTGGQRPSGTGSLQVTVQSGDTMSGIAARYGA
1jfx.1.A       ............................................................

                    350       360       370       380       390       400
020402_00271   WPLSSWSVPSGNLNLIYPGQVVTYNGGGSVATGSNAPPATRTVTVRSGDTLSGIAARLGI
1jfx.1.A       ............................................................

                    410       420
020402_00271   GYTQLTGYRSGNPNVIYPGEVLHY
1jfx.1.A       ........................
```

**Figure S4.** Amino acids sequence of 020402_00271 protein.

MDIKNKSKPNLVTRILAVIVAVLLGVSPATAVADMQGIDVSSWQPSNITRLVDA
DLAVVKVTQATGYVNPSWRAQAQGAVDTGKALGLYHYAGGYNATSEADWF
LAQIGDYVGRAVLVLDWESNQNSAWGNGGWVLQFVQRIHDRTRVWPLVYV
QASAIGQIPQQVWDKCGLWVAQYANMLPTGYQSNPWNYGAYGEAMRQYTS
TGRLPGYSGNLDLNVFRGERWQWDAYAQGDGAKRPSAPLPETTTGATGQQT
ANTGRQCVVVQPGDSLSAIAARYGGTWGEWTGYASGNPGLIYAGETVCRGT
TQQTVTGGQRPSGTGSLQVTVQSGDTMSGIAARYGAWPLSSWSVPSGNLNLI
YPGQVVTYNGGGSVATGSNAPPATRTVTVRSGDTLSGIAARLGIGYTQLTGYR
SGNPNVIYPGEVLHY

**Figure S5.** Analysis of conserved sites of 020402_LYZ M1.

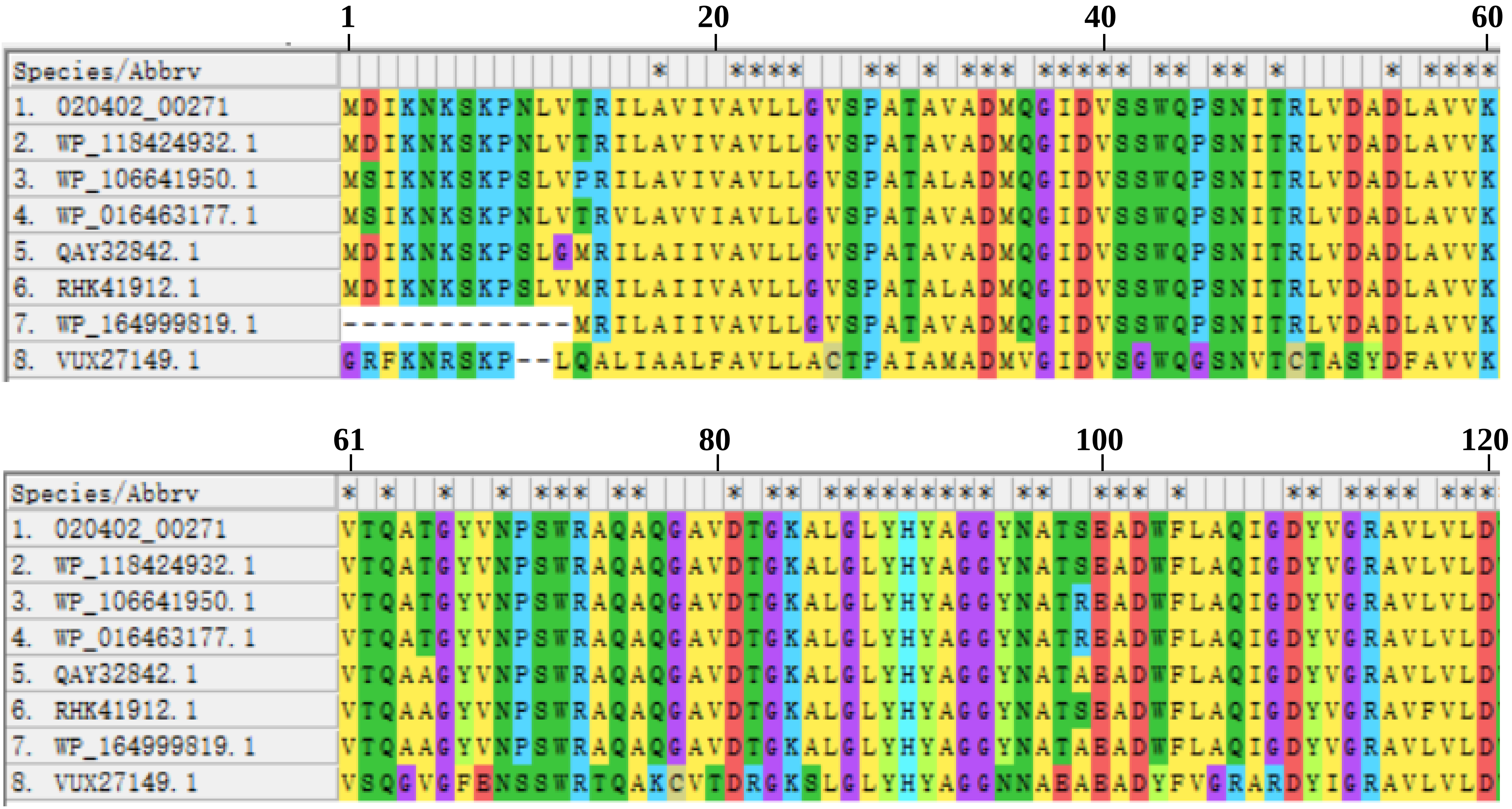

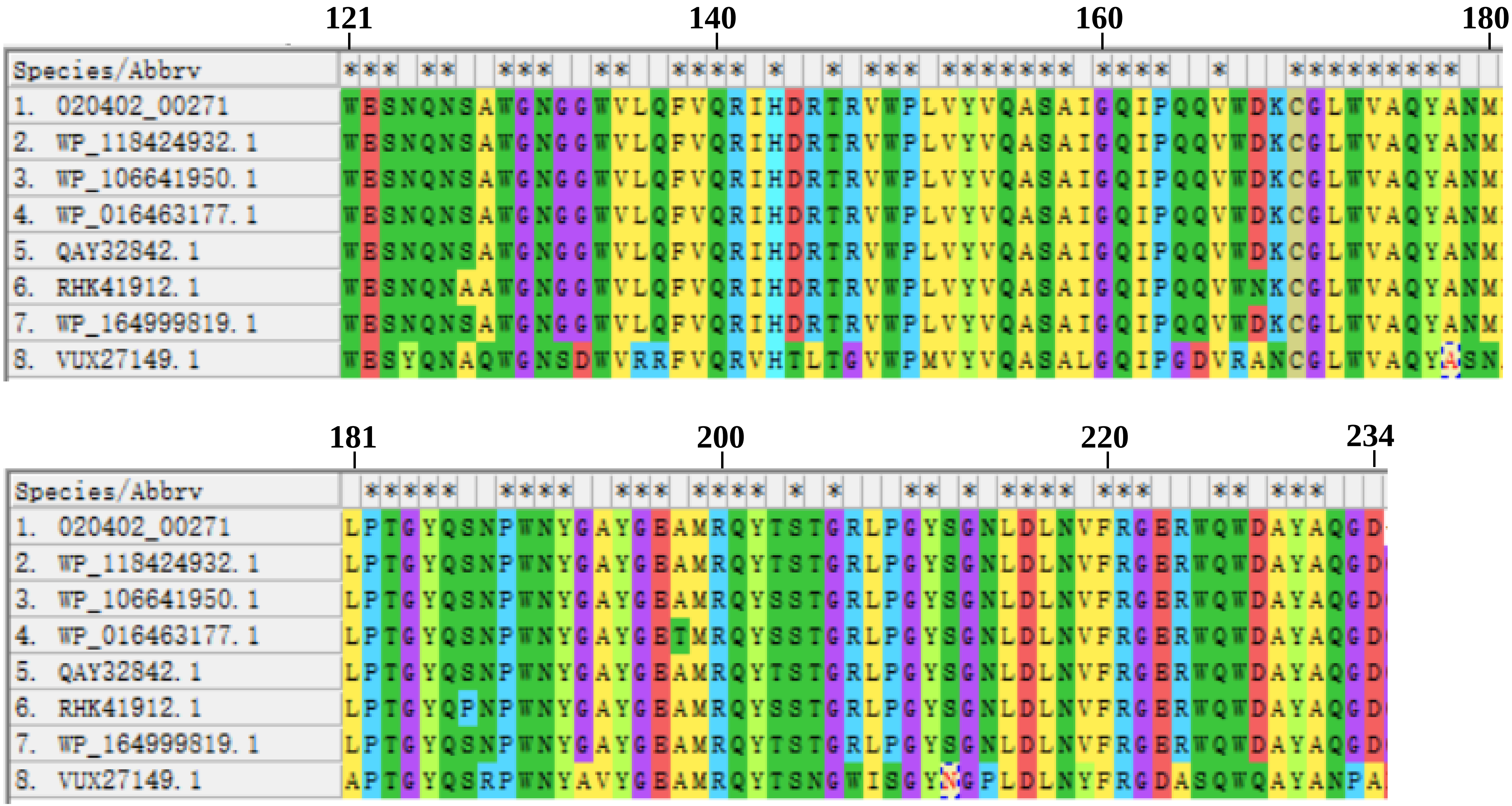
121
140
160
180
181
200
220
234
Species/Abbrv
1. 020402_00271
2. WP_118424932.1
3. WP_106641950.1
4. WP_016463177.1
5. QAY32842.1
6. RHK41912.1
7. WP_164999819.1
8. VUX27149.1
